# Characterization of *Arabidopsis* Post-Glycosylphosphatidylinositol Attachment to Proteins Phospholipase 3 Like Genes

**DOI:** 10.3389/fpls.2022.817915

**Published:** 2022-02-11

**Authors:** Cesar Bernat-Silvestre, Yingxuan Ma, Kim Johnson, Alejandro Ferrando, Fernando Aniento, María Jesús Marcote

**Affiliations:** ^1^Departamento de Bioquímica y Biología Molecular, Instituto Universitario de Biotecnología y Biomedicina (BIOTECMED), Universitat de València, Valencia, Spain; ^2^School of BioSciences, The University of Melbourne, Parkville, VIC, Australia; ^3^Department of Animal, Plant and Soil Sciences, La Trobe Institute for Agriculture and Food, La Trobe University, Bundoora, VIC, Australia; ^4^Instituto de Biología Molecular y Celular de Plantas, Consejo Superior de Investigaciones Científicas, Universitat Politècnica de València, Valencia, Spain

**Keywords:** Glycosylphosphatidylinositol (GPI), GPI-anchored proteins, Per1p, PGAP3, lipid remodeling, *Arabidopsis*

## Abstract

Lipid remodeling of Glycosylphosphatidylinositol (GPI) anchors is required for their maturation and may influence the localization and function of GPI-anchored proteins (GPI-APs). Maturation of GPI-anchors is well characterized in animals and fungi but very little is known about this process in plants. In yeast, the GPI-lipid remodeling occurs entirely at the ER and is initiated by the remodeling enzyme Bst1p (Post-Glycosylphosphatidylinositol Attachment to Proteins inositol deacylase 1 -PGAP1- in mammals and *Arabidopsis*). Next, the remodeling enzyme Per1p (Post-Glycosylphosphatidylinositol Attachment to Proteins phospholipase 3 -PGAP3- in mammals) removes a short, unsaturated fatty acid of phosphatidylinositol (PI) that is replaced with a very long-chain saturated fatty acid or ceramide to complete lipid remodeling. In mammals, lipid remodeling starts at the ER and is completed at the Golgi apparatus. Studies of the *Arabidopsis PGAP1* gene showed that the lipid remodeling of the GPI anchor is critical for the final localization of GPI-APs. Here we characterized loss-of-function mutants of *Arabidopsis Per1*/*PGAP3* like genes (*AtPGAP3A* and *AtPGAP3B*). Our results suggest that *PGAP3A* function is required for the efficient transport of GPI-anchored proteins from the ER to the plasma membrane/cell wall. In addition, loss of function of *PGAP3A* increases susceptibility to salt and osmotic stresses that may be due to the altered localization of GPI-APs in this mutant. Furthermore, *PGAP3B* complements a yeast strain lacking *PER1* gene suggesting that PGAP3B and Per1p are functional orthologs. Finally, subcellular localization studies suggest that PGAP3A and PGAP3B cycle between the ER and the Golgi apparatus.

## Introduction

GPI-anchored proteins (GPI-APs) are involved in diverse and crucial biological processes, including growth, morphogenesis, reproduction, and disease pathogenesis (Cheung et al., 2014). The GPI anchor is newly synthesized in the ER and is then attached to the protein (also synthesized in the ER) by a GPI transamidase (Desnoyer et al., 2020; Kinoshita, 2020). The nascent protein has a N-terminal secretory peptide and a C-terminal GPI-specifying hydrophobic signal sequence where the GPI anchor will be attached (Yeats et al., 2018). The structure of the GPI anchor is conserved in many eukaryotes and it has a common backbone with a glycan core structure and a lipid moiety composed of phosphatidylinositol (PI). Once the GPI anchor is transferred onto the protein at the ER, the glycan core and the lipid moiety need to be remodeled to the mature form of the GPI anchor which is present in the GPI-APs located at the plasma membrane. The mature GPI anchor structures differ between mammals and yeast. The PI form of mature yeast GPI anchors contains either diacylglycerol (DAG) with a very long chain saturated fatty acid (C26:0) at the sn-2 position or ceramide containing phytosphingosine with a very long chain (C26:0) fatty acid (Kinoshita and Fujita, 2016). In contrast, the major form of mammalian mature GPI anchors has 1-alkyl-2-acyl PI bearing a sn2-linked saturated fatty acid (usually stearic acid) (Kinoshita and Fujita, 2016). In plants, only a single GPI anchor structure has been resolved, the one of PcAGP1, isolated from *Pyrus communis* (pear) cell suspension culture. The lipid moiety of PcAGP1 consists of a ceramide, as has been detected in yeast (Oxley and Bacic, 1999). A ceramide was also detected as the lipid component of the GPI anchor of an arabinogalactan protein (AGP) isolated from *Rosa* sp. cell suspension culture (Svetek et al., 1999).

**FIGURE 1:**
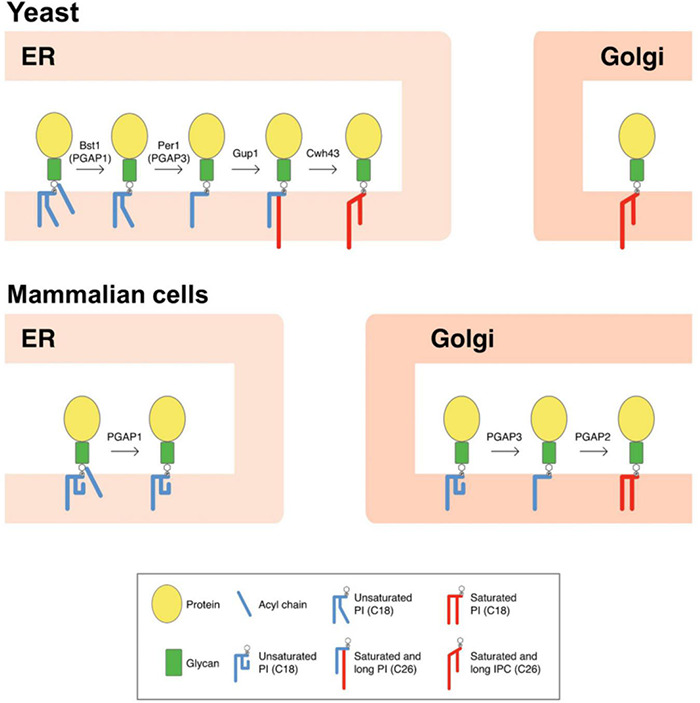
Scheme of GPI-lipid remodelingin yeast and mammalian cells. The GPI anchor is synthesized in the ER and consists of a glycan core and phosphatidylinositol (PI) with an acyl chain at the two position of the inositol ring (Kinoshita and Fujita, 2016). After protein attachment, the glycan and lipid moieties are remodeled sequentially and this process has been shown to be very important for the transport and final localization of the GPI-APs. The GPI-lipid undergoes a structural remodeling that has the purpose of providing saturated lipids and it is initiated at the ER with the inositol deacylation at the two position of the inositol ring by the remodeling enzyme Bst1p (PGAP1 in mammals). Next, the short and unsaturated fatty acid at the sn-2 position of PI is removed by Per1p (PGAP3 in mammals). In yeast, lipid remodeling of the GPI anchor occurs at the ER. In contrast, in mammals lipid remodeling starts at the ER and is completed at the Golgi. The rest of lipid remodeling enzymes are also indicated and details provided in the text (modified from Muniz and Zurzolo, 2014). Only lipid remodeling enzymes are shown. IPC, inositolphosphoceramide.

The lipid remodeling is a critical process for the transport and correct cellular localization of GPI-APs. In yeast, lipid remodeling of the GPI anchor occurs entirely at the ER (Figure 1; Pittet and Conzelmann, 2007). This route is initiated with the enzyme Bst1p, which carries out the deacylation at the two position of the inositol ring (Tanaka et al., 2004), making GPI-APs sensitive to bacterial phosphatidylinositol phospholipase C (PI-PLC). Next, the short and unsaturated fatty acid (C18:1) at the sn-2 position of PI is removed by the Per1p enzyme (Fujita et al., 2006a), and then it is replaced with a very long-chain saturated fatty acid (C26:0) by the membrane-bound O-acyltransferase Glycerol uptake 1 (Gup1p; Bosson et al., 2006). Only those GPI-APs destined to be released to the cell wall seem to maintain the C26:0 DAG generated. Indeed, GPI-APs destined to remain at the plasma membrane contain a ceramide moiety (instead of DAG) consisting of phytosphingosine with a C26 fatty acid. Calcofluor white-hypersensitive 43 (Cwh43p) is the enzyme in charge of adding the ceramide (Umemura et al., 2007; Yoko-o et al., 2018). Although the substrate for the ceramide substitution remains elusive, it seems that most lipid moieties of GPI anchors are exchanged from DAG to ceramide types (Ghugtyal et al., 2007).

Once GPI anchor remodeling is completed at the ER, GPI-APs with long-chain saturated fatty acids have different physical properties and associate to form membrane ordered domains at the ER lipid membrane (Silva et al., 2006), being selectively concentrated at specific ER export sites (ERES) different from those containing other secretory proteins (Muñiz and Riezman, 2016). As the GPI-APs are at the luminal side of the ER, they need a cargo receptor to be sorted within COPII vesicles. This function is carried out by the p24 protein complex (Castillon et al., 2011), which interacts with the remodeled glycan core of GPI-APs and incorporates them into nascent COPII vesicles to transport them to the Golgi apparatus. Once in the Golgi, GPI-APs dissociate from the p24 protein complex and continue their transport to reach their final destination, the plasma membrane or the cell wall.

In mammals, inositol deacylation is mediated by Post-Glycosylphosphatidylinositol Attachment to Proteins inositol deacylase 1 (PGAP1) at the ER (Figure 1; Tanaka et al., 2004). Then, an ethanolamine phosphate (EtNP) side branch linked to the glycan core is removed by an EtNP phosphodiesterase called PGAP5 (Fujita et al., 2009). After these two remodeling reactions, GPI-APs associate with the p24 complex to be transported to the Golgi where the lipid remodeling will continue. In contrast to yeast, mammalian p24 proteins are required not only for packaging GPI-APs into COPII-coated vesicles for ER-Golgi transport but also for concentrating them into the ERESs. This difference may reflect the fact that lipid remodeling of GPI-APs in mammals (which determine their final lipid composition), is not completed at the ER, which may prevent lipid-based sorting into ERES. Once at the Golgi, the unsaturated fatty acid at the sn-2 position of PI of mammalian GPI-APs is replaced by a saturated fatty acid, usually stearic acid. The removal of the unsaturated fatty acid is mediated by the functional ortholog of Per1p, the Golgi enzyme called PGAP3 (Figure 1; Maeda et al., 2007). PGAP3 and Per1p seem to be GPI specific phospholipases A2, although direct demonstration of having this enzyme activity has not been obtained (Pei et al., 2011). The Golgi-resident membrane protein PGAP2 is the enzyme required for the reacylation of the lysoPI with stearic acid (Tashima et al., 2006). Once the GPI anchor is correctly remodeled, some GPI-anchored proteins can also transiently homodimerize (Suzuki et al., 2012) and associate with membrane microdomains or lipid rafts (membrane domains rich in sphingolipids and sterols) (Brown and Rose, 1992; Simons and Gerl, 2010; Zurzolo and Simons, 2016), to be sorted to the apical plasma membrane in polarized cells (Paladino et al., 2006).

In *Arabidopsis thaliana*, around 300 proteins have been predicted to be GPI-APs and among them there are cell wall structural proteins, proteases, enzymes, receptor-like proteins (RLPs), and lipid transfer proteins. They play important roles in a variety of plant biological processes, including cell wall synthesis, polar cell expansion, stress and hormone signaling responses, stomatal development and pollen tube elongation (Yeats et al., 2018; Zhou, 2019). Complete disruption of GPI-anchor synthesis in *Arabidopsis* is lethal, as is the case in yeast and mammals (Lalanne et al., 2004; Gillmor et al., 2005; Dai et al., 2014; Bundy et al., 2016), indicating the vital role of these proteins. Disruption of GPI-anchor lipid remodeling catalyzed by Bst1/PGAP1 or Per1p/PGAP3 is not lethal, neither in yeast (Elrod-Erickson and Kaiser, 1996; Fujita et al., 2006a,b) nor in mammals (Ueda et al., 2007; Murakami et al., 2014; Williams et al., 2015; Kinoshita, 2020). Recently, we found that AtPGAP1 is an ER protein involved in deacylation of the inositol ring of GPI-APs in *Arabidopsis* and this process was shown to be important for the transport and final subcellular localization of GPI-APs. In this work, we have used a loss-of-function approach to initiate the study of the role of *Arabidopsis* orthologs of mammalian PGAP3 and yeast Per1p, the enzymes involved in the removal of the unsaturated fatty acid at the sn-2 position of the GPI-anchor of GPI-APs.

## Materials and Methods

### Plant Material

*Nicotiana benthamiana* plants were grown from surface-sterilized seeds on soil in the greenhouse at 24°C with 16 h daylength. *A. thaliana* plants were grown in growth chambers as previously described (Ortiz-Masia et al., 2007) and ecotype Col-0 was used as wild-type. *Arabidopsis pgap3A* T-DNA insertion mutants used in this study were obtained from the Nottingham Arabidopsis Stock Centre. The T-DNA insertion mutants were characterized by PCR (Supplementary Table 1). Due to the lack of *PGAP3B* T-DNA insertion mutants in mutant collections, artificial microRNA (amiRNA) was used to knock-down the expression of this gene. The *PGAP3B* amiRNA construct CSHL_013451 was purchased from Arabidopsis Biological Resource Center (ABRC)^1^. This construct contained an amiRNA (that we called *amiR-PGAP3B*) that is targeted to a sequence of the last exon of PGAP3B. After transformation with this construct, transgenic plants were selected by antibiotics and segregation of these lines were analyzed. T3 homozygous generation was used to characterize silencing by RT-PCR. Two independent homozygous lines, *amiR-pgap3B-1* and *amiR-pgap3B-2*, that showed the best silencing for PGAP3B were selected. *pgap3A-1* plants were transformed with the *amiR-PGAP3B* construct to generate *amiR-pgap3Bpgap3A* double mutants.

To study whether salt tolerance was affected in the *AtPGAP3* mutants, seeds of wild-type (Col-0) and mutants were sown on Murashige and Skoog (MS) plates containing 160 mM NaCl. Plates were transferred to a controlled growth chamber after cold treatment in the dark for 3 days at 4°C. After 12 days, the rates of cotyledon greening were scored (Sánchez-Simarro et al., 2020). To study mannitol (300 mM) and MgCl_2_ (25 mM) tolerance the same protocol was used, but in the case of MgCl_2,_ seedling survival was scored after 18 days.

### RT-PCR

Total RNA was extracted from seedlings by using a Qiagen RNeasy plant mini kit, and 3 μg of the RNA solution was reverse-transcribed using the maxima first-strand cDNA synthesis kit for quantitative RT-PCR (Fermentas^®^, Canada) according to the manufacturer’s instructions. Semi-quantitative PCRs (sqPCRs) were performed on 3 μl of cDNA template using Emerald Amp Max PCR Master Mix (Takara^®^, Japan). The sequences of the primers used for PCR amplifications are included in Supplementary Table 2.

### Constructs and Antibodies

The coding sequence of PGAP3A-RFP, GFP-PGAP3A, PGAP3B-RFP, GFP-PGAP3B, and GFP-PER1p were commercially synthesized *de novo* (Geneart AG^®^, Germany) based on the sequence of *PGAP3A* (AT5G62130.2), *PGAP3B* (AT1G16560.1), *PER1* (YCR044C), RFP and GFP. For the N-terminal tagged constructs, the GFP cDNA was located after the predicted signal peptide sequence. As the representative model gene of *PGAP3A*, AT5G62130.2, does not include a signal peptide sequence, for the N-terminal GFP-PGAP3A construct, the AT5G62130.1 gene variant was chosen. All the coding sequences were cloned into pCHF3 (pro35S) (Ortiz-Masia et al., 2007). Constructs for yeast expression were obtained as follows: *BamHI*-*SalI* inserts containing either GFP or RFP-tagged *PGAP3A* and *PGAP3B* previously cloned into pCHF3 were subcloned as *BamHI*-*SalI* fragments into pYPGE15 yeast expression vector (Brunelli and Pall, 1993).

A pGreenII 0179 vector backbone (Hellens et al., 2000) was used for constructing V-FLA11 driven by pro35S as previously described (Bernat-Silvestre et al., 2021b). Other constructs used for transient expression experiments were: GFP-AGP4, GFP-GPI, MAP-GFP, and GFP-PAP (Martinière et al., 2012; Bernat-Silvestre et al., 2020, 2021b), GFP-PMA (Kim et al., 2001), PIP2A-RFP (Nelson et al., 2007), RFP-calnexin (Künzl et al., 2016), and GFP-CESA3 (Bernat-Silvestre et al., 2021b). Other constructs have been described previously: RFP−p24δ5 (Langhans et al., 2008; Montesinos et al., 2012), ManI-YFP and ManI-RFP (Nebenführ et al., 1999), ST-YFP (Boevink et al., 1998), GFP-HDEL (Pain et al., 2019), mCherry-HDEL (Nelson et al., 2007), OsSCAMP1-YFP (Lam et al., 2007), GFP-EMP12 (Gao et al., 2012), TIP1.1-GFP (Gattolin et al., 2011), and SPΔCt-mCherry (Pereira et al., 2013).

### Yeast Growth and Complementation

Wild-type yeast strain BY4742 and the isogenic *per1* knock-out mutant were obtained from EUROSCARF with accession numbers Y10000 and Y15768, respectively. The received strains were grown in standard YPD medium. The mutant strain *per1* was transformed with *GFP* or *RFP*-tagged *PGAP3A* and *PGAP3B* constructs in pYPGE15 and selected by *URA3* selectable marker in synthetic SD medium supplemented with histidine, lysine and leucine following the lithium acetate method (Ito et al., 1983). Yeast culture conditions were as described previously (Ferrando et al., 1995). For the drop tests, stationary cultures grown for 2–3 days in either rich medium for the wild type and isogenic per1 mutant or in synthetic SD medium without uracil for the *per1* transformants, were either directly spotted (5 μL) on the plates or serially diluted ×5 fold in the same medium prior to being spotted on the plates.

### Transient Gene Expression in *Arabidopsis* Protoplasts, *Arabidopsis* Seedlings and *Nicotiana benthamiana* Leaves

To obtain mesophyll protoplasts from *Arabidopsis* plants, the Tape-Arabidopsis Sandwich method was used, as described in Wu et al. (2009). Protoplasts were isolated from 4-week old rosette leaves. For transient expression, we used the PEG transformation method (Yoo et al., 2007). Transient expression of *Arabidopsis* seedlings by vacuum infiltration (Bernat-Silvestre et al., 2021a) and *N. benthamiana* leaves mediated by *Agrobacterium tumefaciens* (Lerich et al., 2011) were performed as described previously.

### Preparation of Protein Extracts and SDS-PAGE and Immunoblotting

*Nicotiana benthamiana* leaves expressing XFP-Proteins were frozen in liquid N_2_ and then ground in homogenization buffer (HB, 0.3 M sucrose; 1 mM EDTA; 20 mM KCl; 20 mM HEPES pH 7.5), supplemented with 1 mM DTT and a Protease Inhibitor Cocktail (Sigma^®^, United States), using a mortar and a pestle. The homogenate was centrifuged for 10 min at 1,200 × *g* and 4°C, and the post nuclear supernatant (PNS) was collected and analyzed by SDS-PAGE and immunoblotting with GFP/RFP antibodies from Rockland Immunochemicals^®^ (United States). For yeast protein extracts, culture cells were pelleted and resuspended in SDS-PAGE sample buffer. Immunoblots were developed using the SuperSignal West Pico chemiluminescent substrate (Pierce, Thermo Fisher Scientific^®^, United States) and analyzed using the ChemiDoc XRS+ imaging system (Bio-Rad^®^, United States)^2^. Immunoblots in the linear range of detection were quantified using Quantity One software (Bio-Rad Laboratories^®^).

### Confocal Microscopy

Confocal fluorescent images were collected using an Olympus FV1000^®^ confocal microscope with 60× oil lens. The GFP signal was visualized with laser excitation at 488 nm and emission at 496–518 nm. The YFP signal was visualized with laser excitation at 514 nm and emission at 539–561 nm. The mRFP/mCherry signal was visualized with laser excitation at 543 nm and emission at 593–636 nm. Sequential scanning was used to avoid any interference between fluorescence channels. Post-acquisition image processing was performed using the FV10-ASW 4.2 Viewer^®^ and ImageJ^®^ (v.1.45).

### Statistical Analysis

Differences in stress responses among *pgap3A*, *pgap3B*, and *pgap3AB* mutants compared to Col-0 (Wild-type) were tested using a two samples *t*-test with unequal variances using Microsoft Excel^®^ 2013.

## Results

### *PGAP3* Genes

The lipid remodeling reaction that removes an unsaturated acyl chain at the *sn-2* position of the PI moiety is mediated by mammalian PGAP3 and yeast Per1p (Figure 1). Both enzymes belong to the membrane bound hydrolase CREST (alkaline ceramidase, PAQR receptor, Per1, SID-1, and TMEM8) superfamily (Pei et al., 2011). Members of this superfamily share seven predicted core transmembrane segments and a set of conserved serine, histidine, and aspartate residues (Supplementary Figure 1). Two *Arabidopsis* genes, AT5G62130 and AT1G16560, have been assigned to belong to the Per1/PGAP3 family of fatty acid remodeling hydrolases for GPI-anchored proteins (Pei et al., 2011). They share 60% amino acid sequence identity and both conserve yeast histidines 177 and 326 that have been shown important for the putative function of Per1 proteins (Fujita et al., 2006a; Pei et al., 2011; Supplementary Figure 1). From now on, AT5G62130 and AT1G16560 will be referred as *PGAP3A* and *PGAP3B*, respectively. *PGAP3A* and *PGAP3B* are predicted to encode a 343-amino acid and 342-amino acid membrane protein, respectively, with an expected subcellular localization at the ER, Golgi apparatus or plasma membrane (Hofmann and Stoffel, 1993)^3^. Transmembrane topology prediction CCTOP (Dobson et al., 2015) suggests that both proteins have an amino-terminal secretory signal peptide and seven transmembrane domains, as occurs in other members of the Per1 family (Supplementary Figure 1). The cytosolic tail of both PGAP3A and PGAP3B contain a C-terminal dilysine motif which has been shown to be involved in the retrieval of proteins from post ER-membranes to the ER (Gao et al., 2014; Supplementary Figure 1).

To investigate the relative expression of *PGAP3* genes, we used the publicly available RNAseq expression database GENEVESTIGATOR (Zimmermann et al., 2004; Hruz et al., 2008). As shown in Supplementary Figure 2, both genes show expression in most tissues throughout plant development with *PGAP3B* (AT1G16560) having higher mRNA transcript levels than *PGAP3A* (AT5G62130).

**FIGURE 2:**
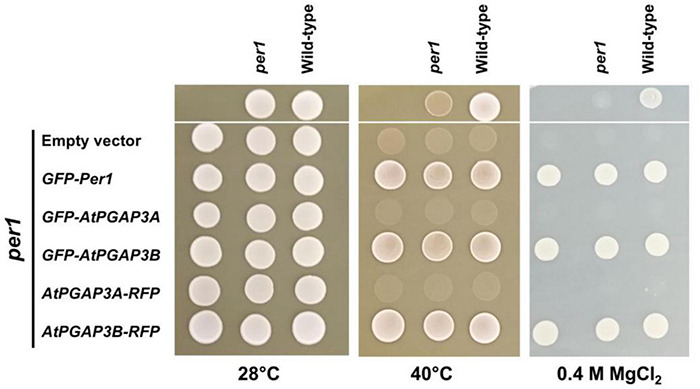
Complementation analysis of the yeast *per1* mutant by *Arabidopsis PGAP3A/B*. Wild-type and *per1* (Y15768) mutant strains were grown in YPD medium for 2 days and spotted (upper part) on the indicated YPD medium and temperatures. In parallel, three independent transformants of *per1* (lower part) with each of the tagged *PGAP3A/B* complementation constructs and *GFP-Per1* and empty vector as controls (as shown on the left) were grown for 2 days in synthetic medium supplemented with the required amino acids and spotted on the same plates and temperature as indicated. Growth was scored after 2 days. The *GFP-Per1* and both *PGAP3B* constructs were able to recover heat and MgCl_2_ sensitivity of the *per1* mutant whereas *PGAP3A* constructs were not.

It has been previously described that *per1* yeast cells showed increased heat and MgCl_2_ sensitivity (Paidhungat and Garrett, 1998; Fujita et al., 2006a). To determine if *PGAP3A* and *PGAP3B* are functional orthologs of *PER1*, we introduced plasmids encoding N-terminal GFP or C-terminal RFP tagged PGAP3A and PGAP3B into yeast *per1* cells. Tagged proteins of the expected molecular weight were detected in yeast (Supplementary Figure 3A). We examined the sensitivities of the yeast *per1* mutant and the complemented lines to 0.4 M MgCl_2_ and high temperature compared to wild-type cells. We found that both the N-terminal as well as the C-terminal *PGAP3B* constructs restored MgCl_2_ and high temperature tolerance of *per1* cells to wild-type levels, as it was the case for *GFP-Per1p* (Figure 2). In contrast, *per1* lines complemented with *PGAP3A* constructs remained sensitive to MgCl_2_ and high temperature. In addition, the *per1* yeast cells were also shown to have a mild phenotype in the presence of 1 M NaCl that could be restored by *PGAP3B* but not by *PGAP3A constructs* (Supplementary Figure 3C).

### Subcellular Localization of PGAP3A and PGAP3B

**FIGURE 3:**
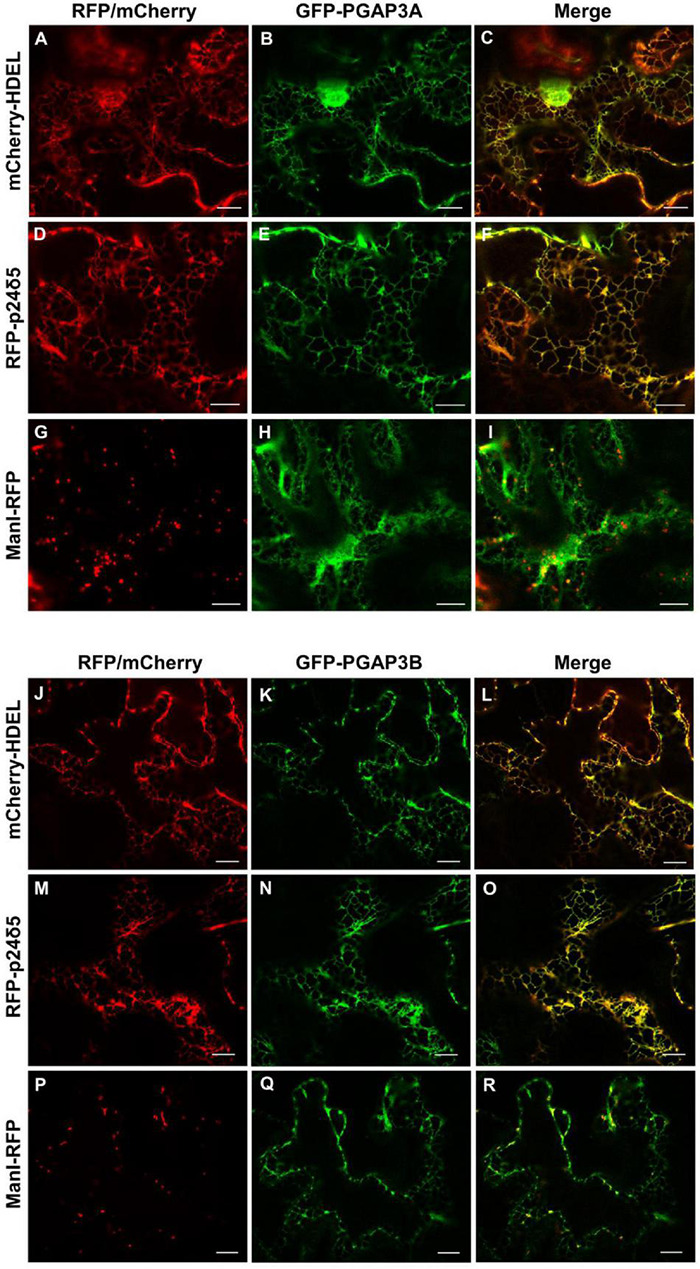
Localization of GFP-PGAP3A and GFP-PGAP3B. Transient expression in *N. benthamiana* leaves of GFP-PGAP3A **(B,E,H)** and GFP-PGAP3B **(K,N,Q)** together with mCherry-HDEL (luminal ER marker) **(A,J)**, RFP-p24δ5 (membrane ER marker) **(D,M)**, and ManI-RFP **(G,P)** (Golgi marker) [see merged images in **(C,F,I,L,O,R)**]. Scale bars = 10 μm.

**FIGURE 4:**
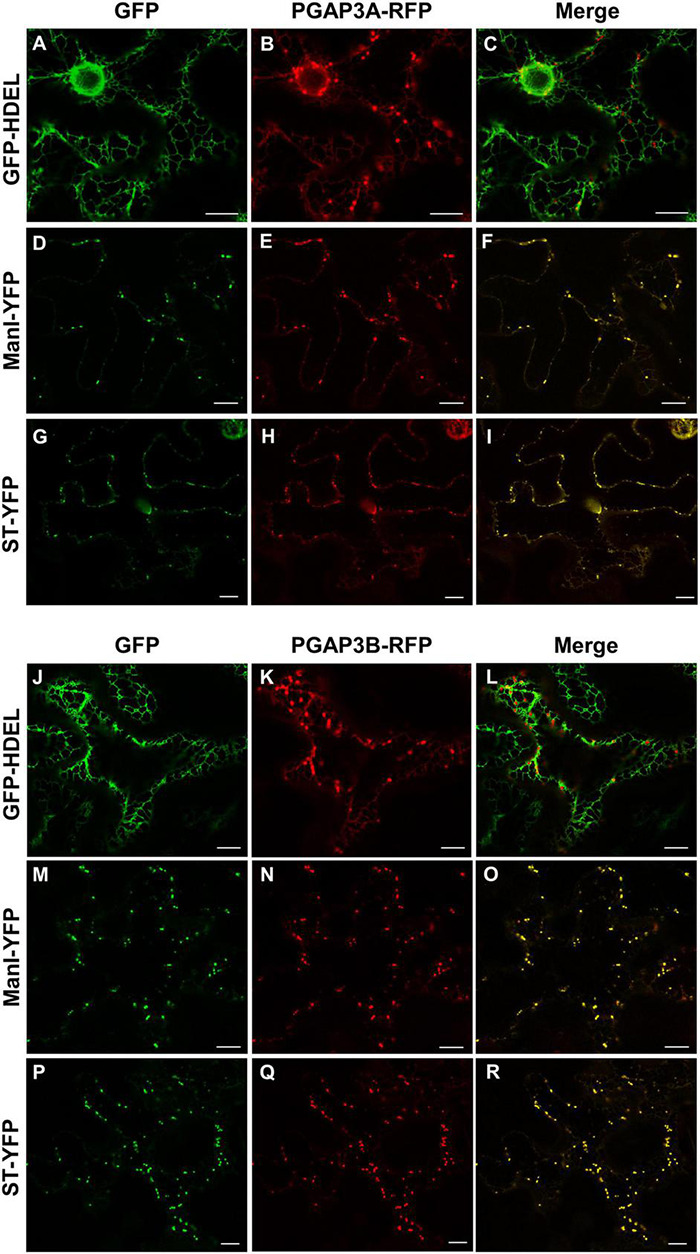
Localization of PGAP3A-RFP and PGAP3B-RFP. Transient expression in *N. benthamiana* leaves of PGAP3A-RFP **(B,E,H)** and PGAP3B-RFP **(K,N,Q)** together with GFP-HDEL (luminal ER marker) **(A,J)**, ManI-YFP **(D,M)**, and ST-YFP **(G,P)** (Golgi markers) [see merged images in **(C,F,I,L,O,R)**]. Scale bars = 10 μm.

As described in the section “Introduction,” yeast Per1p has been proposed to localize at the ER. However, the mammalian ortholog of Per1p, PGAP3, mainly localizes at the Golgi with a minor ER localization (Maeda et al., 2007; Howard et al., 2014). This is consistent with the fact that mammalian GPI-APs are segregated and sorted at the Golgi apparatus (where the lipid remodeling is completed). Therefore, we sought to investigate subcellular localization of the two isoforms of *Arabidopsis* PGAP3. In order to localize PGAP3A-B *in vivo*, PGAP3A and PGAP3B constructs, with N- or C-terminal GFP and RFP, respectively, were used for transient expression in *Nicotiana benthamiana* leaves. Protein extracts were analyzed by SDS-PAGE and Western Blot with GFP and RFP antibodies to confirm that proteins of the expected size were present (Supplementary Figure 3B). As shown in Figure 3, both GFP-PGAP3A and GFP-PGAP3B showed an ER-like localization pattern and extensively colocalized with the ER markers mCherry-HDEL and RFP-p254δ5. Occasionally, GFP-PGAP3B was also found in punctate structures which partially colocalized with the Golgi marker ManI-RFP (Figures 3L,O,R). When RFP was placed at the C-terminus of both proteins, we observed a shift in the localization of PGAP3A-RFP and PGAP3B-RFP. Both proteins showed a punctate pattern and extensively colocalized with the Golgi markers ManI-YFP and ST-YFP, although some ER localization was also detected (Figure 4). Since PGAP3A and PGAP3B both contain a canonical ER retrieval/retention signal at their C-terminus (KKxx in PGAP3A, KxKxx in PGAP3B) (Supplementary Figure 1), the shift in the localization of the C-terminal tagged proteins may be caused by masking of their ER retrieval/retention signals. These results suggest that, irrespective to their steady-state localization, both PGAP3A and PGAP3B may cycle between ER and Golgi.

### Characterization of *pgap3* Mutants

**FIGURE 5:**
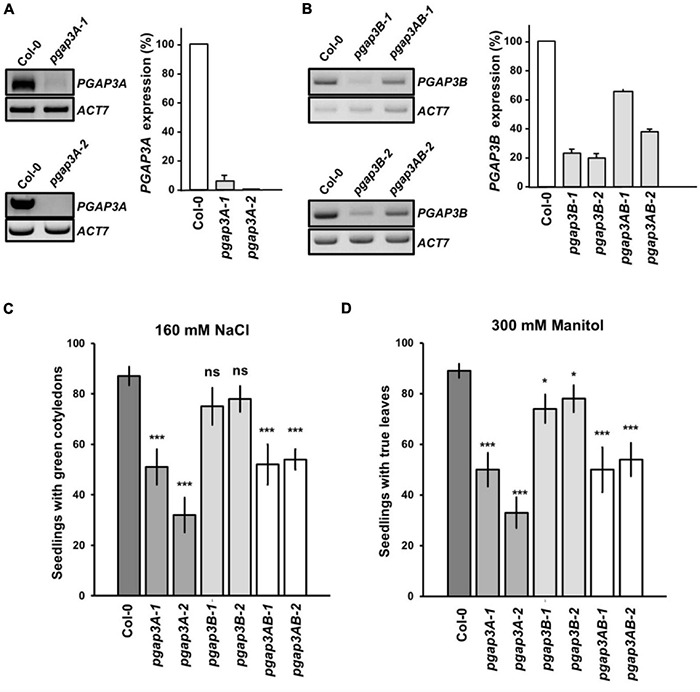
Characterization of *pgap3* mutants. **(A,B)** sqPCR analysis of *PGAP3A* expression in *pgap3A* mutants **(A)** and *PGAP3B* expression in *pgap3B* and *pgap3AB* mutants **(B)**. Total RNA from *pgap3A-1, pgap3A-2, pgap3B-1, pgap3B-2, pgap3AB-1, pgap3AB-2*, and wild-type (Col-0) 4 day-old seedlings were used for the PCRs. In the PCRs, *PGAP3A* and *PGAP3B* specific primers were used (Supplementary Tables 1, 2). *Actin-7* (*ACT7*) was used as a control. PCR samples were collected at cycle 22 for *ACT7*, at cycle 36 for *PGAP3A* and at cycle 25 for *PGAP3B.* No wild-type band was detected in *pgap3A-2.* Quantification of the bands (*n* = 3) are shown. Values were normalized against the *PGAP3A/PGAP3B* fragment band intensity in wild-type that was considered to be 100%. Error bars represent SEM. **(C,D)** Phenotype of *pgap3A*, *pgap3B*, and *pgap3AB* mutants exposed to salt (NaCl) **(C)** and mannitol **(D)**. Wild type (Col-0), *pgap3A-1, pgap3A-2, pgap3B-1, pgap3B-2, pgap3AB-1*, and *pgap3AB-2* mutants were grown on 0.5 × MS as a control and 0.5 × MS supplemented with 160 mM NaCl or 300 mM mannitol for 12 days. The percentage of seedlings with green cotyledons and seedlings with true leaves was calculated for NaCl and mannitol experiments, respectively. Data shown as the mean ± SEM of five independent experiments (*n* = 20). Statistical significance: ns, not significant; *p < 0.05; **p < 0.01; ***p < 0.001.

*Arabidopsis* T-DNA insertion mutants were characterized to further study *PGAP3A* and *PGAP3B* function. Two *PGAP3A* T-DNA insertion mutants from the SALK collection^4^, *pgap3A-1* (SALK_039375), and *pgap3A-2* (SALK_069053), were characterized (Figure 5 and Supplementary Figure 4). The mRNA levels of *PGAP3* in *pgap3A-1* were less than 10% of wild-type levels and no *PGAP3* mRNA could be detected in *pgap3A-2* by RT-PCR analysis (Figure 5). These results indicate that *pgap3A-1* and *pgap3A-2* are knock-down and knock-out mutants, respectively. Due to the lack of *PGAP3B* T-DNA insertion mutants in mutant collections, an artificial microRNA (*amiR-PGAP3B*) was used to knock-down the expression of this gene (Supplementary Figure 4). *A. thaliana* transgenic lines were generated by transformation with *amiR-PGAP3B*. Independent lines were selected and the T3 homozygous generation was used to characterize silencing by RT-PCR as above. Two independent homozygous lines, *amiR-pgap3B-1* and *amiR-pgap3B-2*, that showed the best silencing for *PGAP3B* (around 20% wild-type mRNA levels) were selected (Figure 5) and from now on, they will be referred as *pgap3B-1* and *pgap3B-2*, respectively. *pgap3A-1* plants were transformed with the *amiR-PGAP3B* construct to generate an *amiR-pgap3Bpgap3A* double mutant. Independent transgenic lines were selected and the T3 homozygous generation was used to characterize silencing by RT-PCR as above. Two independent homozygous lines, *amiR-pgap3Bpgap3A-1* and *amiR-pgap3Bpgap3A-2*, that showed the best silencing for *PGAP3B* (less than 70 and 40% of mRNA levels, respectively) were selected (Figure 5) and from now on, they will be referred as *pgap3AB-1* and *pgap3AB-2*, respectively.

None of the single mutants of *PGAP3A*, *PGAP3B* nor the double mutants of *PGAP3AB* showed any obvious phenotypic alteration under standard growth conditions when compared to wild-type plants (Supplementary Figure 4). However, we found that *pgap3A-1* and *pgap3A-2* showed enhanced sensitivity to 160 mM NaCl and 300 mM mannitol. The same sensitivity was observed in *pgap3AB* double mutants (Figures 5C,D). Interestingly, *pgap3A* and *pgap3AB* mutants were also more sensitive than wild-type to 25 mM MgCl_2_ (Supplementary Figure 4F) as yeast *per1* cells. In general, smaller differences were detected between *pgap3B* mutants and wild-type in all the sensitivities tested (Figure 5 and Supplementary Figure 4).

### Localization of GPI-Anchored Proteins in *pgap3* Mutants

Lipid remodeling enzyme function has been shown to be important for the efficient transport from the ER to the plasma membrane of yeast, mammalian and *Arabidopsis* GPI-anchored proteins (Tanaka et al., 2004; Bernat-Silvestre et al., 2021b). For that reason, we analyzed the localization of two GPI-anchored proteins in *pgap3* mutants. One of them was GFP fused to arabinogalactan protein 4 (AGP4), a GPI-AP proteoglycan that seems to be involved in diverse developmental processes (Ellis et al., 2010; Pereira et al., 2016). This protein was shown previously to localize to the plasma membrane/apoplast (Martinière et al., 2012; Bernat-Silvestre et al., 2020, 2021b). The second one was Venus fused to FLA11 (V-FLA11), a member of fasciclin-like arabinogalactan proteins (FLAs) that have been related to cell adhesion (Johnson, 2003; MacMillan et al., 2010). In addition, we also used a glycosylphosphatidylinositol-anchored GFP (GFP-GPI; Martinière et al., 2012; Bernat-Silvestre et al., 2020, 2021b). As a control, we used a transmembrane plasma membrane protein, the aquaporin PIP2A-RFP (Nelson et al., 2007).

**FIGURE 6:**
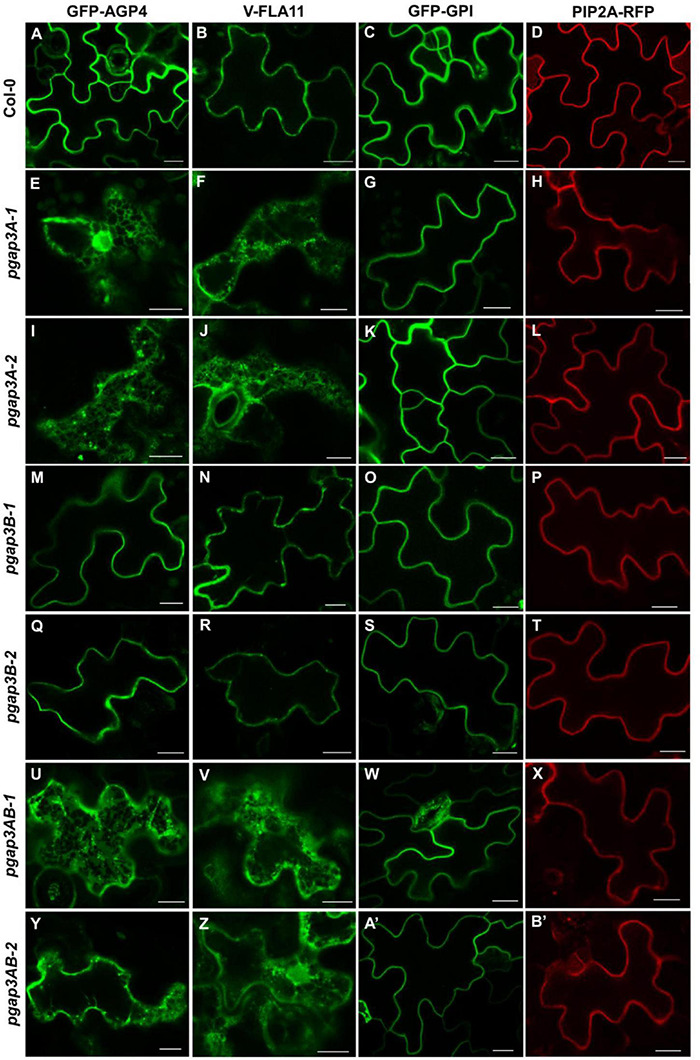
Localization of GFP-AGP4, V-FLA11, GFP-GPI, and PIP2A-RFP in wild-type, *pgap3A*, *pgap3B*, and *pgap3AB Arabidopsis* seedlings. Transient expression of GPI-anchored proteins and a plasma membrane marker in seedlings of wild-type (Col-0) **(A–D)** or *pgap3A-1* **(E–H)**, *pgap3A-2* **(I–L)**, *pgap3B-1* **(M–P)**, *pgap3B-2* **(Q–T)**, *pgap3AB-1* **(U–X)**, and *pgap3AB-2* **(Y–B’)** mutants. The three GPI-anchored proteins, GFP-AGP4, V-FLA11, and GFP-GPI, mainly localized to the plasma membrane in cotyledon cells from wild-type (Col-0), *pgap3B-1*, and *pgap3B-2* mutant seedlings, as the transmembrane protein PIP2A-RFP. In the *pgap3A-1*, *pgap3A-2*, *pgap3AB-1* and *pgap3AB-2* mutants, GFP-AGP4 and V-FLA11 showed a predominant ER localization pattern as well as a punctate pattern, probably corresponding to Golgi structures, in contrast to GFP-GPI and PIP2A-RFP, which mainly localized to the plasma membrane. Scale bars = 10 μm.

We first analyzed the localization of these proteins by transient expression in *Arabidopsis* seedlings (Figure 6; Bernat-Silvestre et al., 2021a). GFP-AGP4, V-FLA11, and GFP-GPI were localized to the plasma membrane/cell wall of cotyledon cells of wild-type *Arabidopsis* seedlings, as it was the case for the transmembrane plasma membrane protein PIP2A-RFP, as shown previously (Bernat-Silvestre et al., 2020, 2021b). In clear contrast, GFP-AGP4 and V-FLA11 showed a predominant ER-like localization pattern, together with a punctate pattern (presumably a Golgi pattern) in the two *pgap3A* mutants and in the two double *pgap3AB* mutants (Figure 6). This was not the case in the *pgap3B* mutants, where both proteins mainly localized to the plasma membrane/cell wall. Interestingly, GFP-GPI localized to the plasma membrane in all mutants, as did the transmembrane protein PIP2A-RFP (Figure 6). This suggests that PGAP3A enzyme is involved in the transport to the plasma membrane of GFP-AGP4 and V-FLA11, and that loss of PGAP3A function does not affect transport from the ER to the plasma membrane of GFP-GPI and the transmembrane protein PIP2A-RFP. The defect in transport of GFP-AGP4 and V-FLA11 in *pgap3A* mutants was not due to an alteration in the compartments of the secretory pathway, since no obvious defects were observed in the localization pattern of several organelle marker proteins, including GFP-HDEL (ER), GFP-EMP12 (Golgi apparatus), TIP1.1-GFP (tonoplast), SPΔCt-mCherry (vacuole lumen), SCAMP1-YFP (plasma membrane), and GFP-CESA3 (TGN/plasma membrane) (Supplementary Figure 5).

**FIGURE 7:**
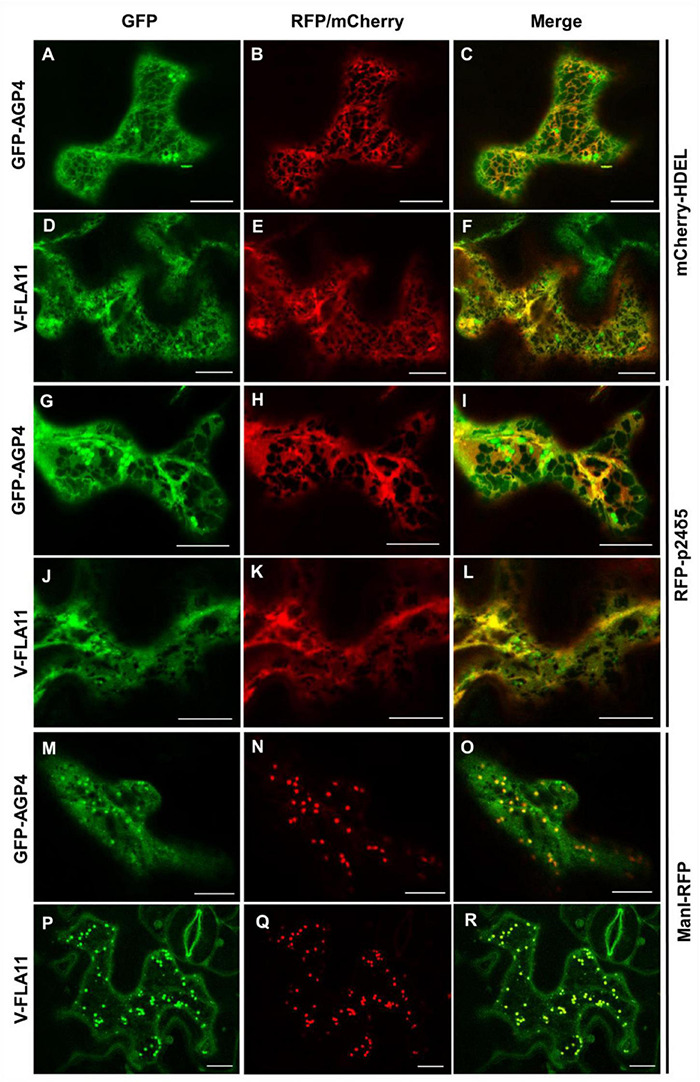
Colocalization of GPI-APs with ER and Golgi markers in *pgap3A-2* seedlings. Transient expression in *Arabidopsis* seedlings. **(A–F)** Coexpression of GFP-AGP4 **(A)** and V-FLA11 **(D)** with the ER marker mCherry-HDEL **(B,E)** [see merged images in **(C,F)**]. **(G–L)** Coexpression of GFP-AGP4 **(G)** and V-FLA11 **(J)** with the ER marker RFP-p24δ5 **(H,K)** [see merged images in **(I,L)**]. **(M–R)** Coexpression of GFP-AGP4 **(M)** and V-FLA11 **(P)** with the Golgi marker ManI-RFP **(N,Q)** [see merged images in **(O,R)**]. Scale bars = 10 μm. GFP-AGP4 and V-FLA11 colocalized with ER marker proteins and they were also found in punctate structures which colocalized with the Golgi marker.

The localization of GFP-AGP4 and V-FLA11 in *pgap3A* mutants was confirmed by colocalization experiments. As shown in Figure 7, both GFP-AGP4 and V-FLA11 strongly colocalized with two different ER marker proteins, an ER luminal protein (mCherry-HDEL) and an ER membrane protein (RFP-p24δ5). In addition, GFP-AGP4 and V-FLA11 were also partially found in punctate structures which colocalized with the Golgi marker ManI-RFP, suggesting that these GPI-anchored proteins also localized to the Golgi apparatus in *pgap3A* mutants.

To confirm these ER/Golgi patterns, we also analyzed the localization of GFP-AGP4 and GFP-GPI by an alternative transient expression system, *Arabidopsis* protoplasts. In protoplasts from wild-type *Arabidopsis* plants, GFP-AGP4 and GFP-GPI localized to the plasma membrane, as we have shown previously (Bernat-Silvestre et al., 2020, 2021b). However, as it happened in transient expression in *Arabidopsis* seedlings, GFP-AGP4 also showed an ER/Golgi localization pattern in protoplasts from the *pgap3A-1* and *pgap3AB-2* mutants, but not in *pgap3B* mutants (Supplementary Figure 6) while GFP-GPI localized to the plasma membrane in all *pgap3* mutants (Supplementary Figure 6). To corroborate the ER/Golgi patterns of GFP-AGP4 in these mutants, we co-expressed GFP-AGP4 with two different ER-membrane markers (RFP-calnexin and RFP-p24δ5) and a Golgi marker (ManI-RFP). As showed in Supplementary Figure 7, these markers extensively colocalized with GFP-AGP4 in *pgap3AB-2* protoplasts, confirming the same ER/Golgi pattern showed in seedlings. Additionally, we could also detect the presence of both GFP-AGP4 and GFP-GPI at the plasma membrane, as shown by colocalization with FM (Fei Mao) styryl dye FM4-64, a lipid probe routinely used to label the plasma membrane (Supplementary Figure 7). This suggests that a fraction of GFP-AGP4 can reach the plasma membrane in *pgap3AB* mutants.

To test if the lack of PGAP3 enzymes affects the localization of other plasma membrane proteins different from GPI-APs, we used plasma membrane markers without a GPI anchor, including a myristoylated and palmitoylated GFP (MAP-GFP), a prenylated GFP (GFP-PAP) (Martinière et al., 2012) and a transmembrane protein, a GFP fusion with the plasma membrane ATPase (GFP-PMA; Kim et al., 2001). As shown in Supplementary Figure 8, these three proteins mainly localized to the plasma membrane in *pgap3A-1, pgap3B-2* and *pgap3AB-2* protoplasts, as in protoplasts from wild-type *Arabidopsis* plants, suggesting that the transport of other plasma membrane proteins is not affected in these mutants.

### Transport of GFP-AGP4 Is Delayed in *pgap3a* Mutants

**FIGURE 8:**
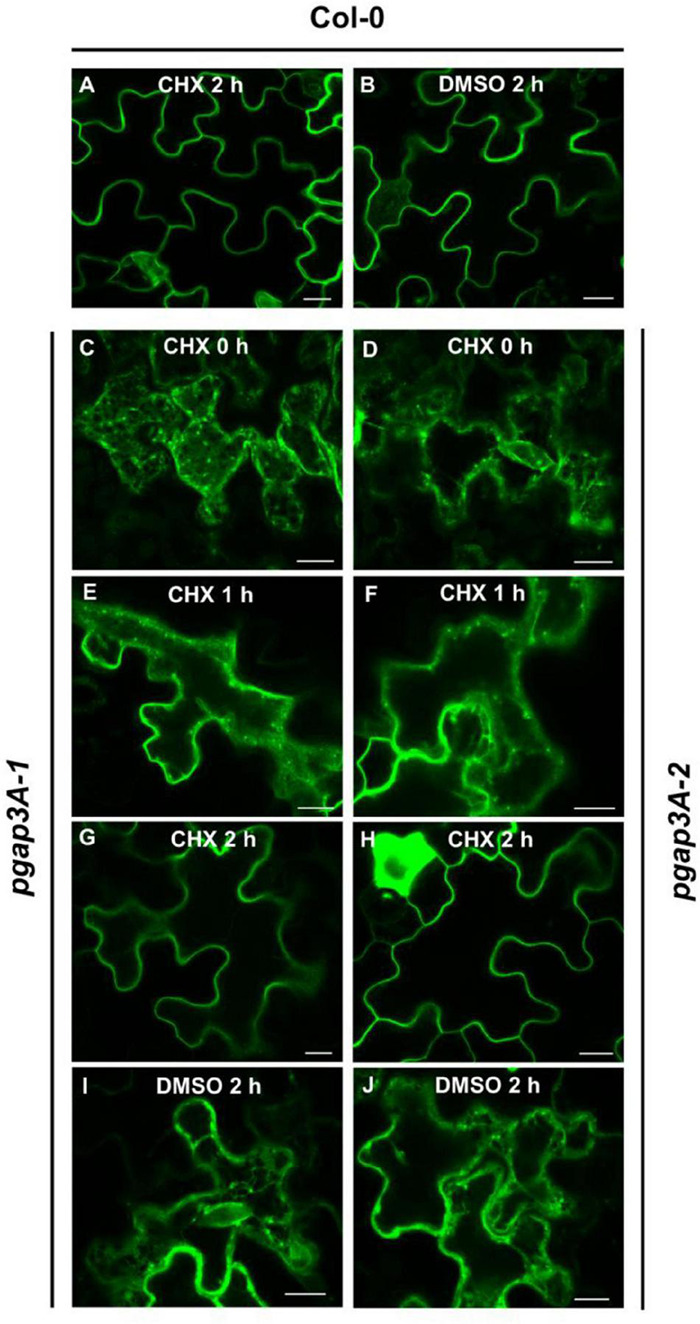
Localization of GFP-AGP4 in wild-type and *pgap3A Arabidopsis* seedlings treated with cycloheximide (CHX). **(A–J)** Transient expression of GFP-AGP4 in *Arabidopsis* seedlings of wild-type (Col-0) **(A,B)**, *pgap3A-1* **(C,E,G,I)**, and *pgap3A-2* **(D,F,H,J)**. Seedlings were incubated in the presence of 20 μM cycloheximide or DMSO (control) for 2 h and analyzed by confocal laser scanning microscopy (CLSM). Scale bars = 10 μM. Note that transport of GFP-AGP4 is delayed in *pgap3A-1* and *pgap3A-2* mutants and GFP-AGP4 can reach the plasma membrane after 2 h in the presence of CHX.

Since GFP-AGP4 partially localized to the plasma membrane in *pgap3A* mutants, we postulated that loss of *PGAP3A* may cause a delay (rather than a block) in its transport to the plasma membrane. Indeed, by inhibiting protein synthesis with cycloheximide, we have previously shown that loss of function of *PGAP1* caused a delay in the transport of GFP-AGP4 from the ER to the cell surface with a progressive relocalization of GFP-AGP4 from the ER to the cell surface and ER labeling being almost undetectable after 6 h (Bernat-Silvestre et al., 2021b). To show if GFP-AGP4 was also able to reach the cell surface in *pgap3A* over time, the localization of GFP-AGP4 was analyzed after inhibition of protein synthesis. Treatment of *pgap3A* seedlings with 20 μM cycloheximide caused a progressive relocalization of GFP-AGP4 from the ER/Golgi to the cell surface, faster than that observed in *pgap1* seedlings, with ER labeling being almost undetectable after 2 h (Figure 8). This indicates that GFP-AGP4 can reach the cell surface in the absence of PGAP3A but with a delayed kinetics and suggests that PGAP3A is involved in efficient transport of GPI-APs from the ER to the cell surface.

## Discussion

Up to now, only one plant GPI anchor structure has been resolved, the one of PcAGP1, isolated from *Pyrus communis* (pear) cell suspension cultures (Oxley and Bacic, 1999). From this structure, it seems that the core structure of GPI anchors is conserved in plant and non-plant eukaryotes. In addition, a survey of the *Arabidopsis* genome indicates that most of the genes involved in particular steps of GPI anchor assembly and their remodeling have orthologs in *Arabidopsis* (Luschnig and Seifert, 2011). However, it has to be established whether *Arabidopsis* orthologs are functional and whether their function is conserved. Null *Arabidopsis* mutants involved in the biosynthesis and attachment of the GPI anchor showed either gametophytic or embryogenic lethality, indicating that GPI-APs are essential for growth and development in *Arabidopsis* (Lalanne et al., 2004; Gillmor et al., 2005; Dai et al., 2014; Bundy et al., 2016). Recently, we reported for the first time the characterization of *AtPGAP1*, an *Arabidopsis* gene involved in lipid remodeling of the GPI anchor (Bernat-Silvestre et al., 2021b). We found that PGAP1 localizes to the ER and likely functions as the GPI inositol-deacylase that cleaves the acyl chain from the inositol ring of the GPI anchor. Loss of PGAP1 function produced a delayed transport of GPI-APs through the secretory pathway, suggesting that PGAP1 is required for efficient ER export and transport to the cell surface of GPI-APs.

In this study, we have initiated the characterization of *Arabidopsis PGAP3A* and *PGAP3B*, orthologs of yeast *PER1* and mammalian *PGAP3*, which have been proposed to function in the removal of the unsaturated fatty acid at the sn-2 position of the GPI-anchor of GPI-APs, although direct evidence of their hydrolase activity is lacking (Pei et al., 2011). AtPGAP3A and AtPGAP3B, together with Per1p and PGAP3, belong to the Per1 family (Pei et al., 2011). PGAP3B fusion proteins were able to rescue heat and salt sensitivity phenotypes of *per1* yeast cells, indicating that PGAP3B may be functionally equivalent to yeast Per1p. This was not the case of PGAP3A. It is possible that XFP-tagged PGAP3A is not active in yeast (due to a defect in a posttranslational modification or to different splicing isoforms involved) although it cannot be discarded that PGAP3A plays a distinct and/or plant specific role.

No obvious phenotypic differences were observed between *pgap3* mutants and wild-type plants under standard growth conditions. Nevertheless, *pgap3A* mutants showed enhanced sensitivity to NaCl, MgCl_2_, and mannitol. This may be due to defects in the localization/concentration of GPI-APs in membrane domains. In MDCK cells, correct lipid remodeling is necessary for proper oligomerization and concentration of GPI-APs in raft microdomains, essential for their transport to the apical or basolateral membranes (Paladino et al., 2004, 2006). Many GPI-APs are signal receptors that function during the response of cells to the extracellular environment (Yeats et al., 2018; Zhou, 2019). Thus, GPI anchor remodeling defects in plants are expected to produce an altered cellular response to salt stress as it has been observed in yeast (Paidhungat and Garrett, 1998; Fujita et al., 2006a). Although PGAP3A did not rescue the salt phenotypes of yeast *per1*, *Arabidopsis pgap3A* mutants also show salt sensitivity. In contrast, *pgap3B* mutants did not show any significant sensitivity to salt and a lower level of sensitivity to mannitol stress than *pgap3A* mutants. Transcript levels of *PGAP3B* are higher than those of *PGAP3A* and are reduced to around 20% of wild-type levels in *pgap3B* mutants. Therefore, the mild phenotypes observed in response to stress in *pgap3B* lines and the lack of an effect on GPI-AP trafficking, suggest that there is enough residual PGAP3 activity in those lines.

In yeast, GPI-AP trafficking was altered in *per1* mutant cells (Fujita et al., 2006a). GPI-APs accumulated at the ER due to inefficient exit from the ER and levels of cell surface GPI-APs (lacking GPI anchor remodeling) were affected. The trafficking of some GPI-APs to the cell surface was also altered in *pgap3A* mutants. Similar to *per1* yeast cells, *pgap3A* mutants showed a delay in the transport of GPI-APs. In mammalian cells, a defect of PGAP3 also results in unremodeled GPI-APs at the cell surface and depending on the proteins, cell types and species, can also affect transport to the cell surface (Maeda et al., 2007, 2017; Kinoshita and Fujita, 2016). Similarly, trafficking of GFP-AGP4 and V-FLA11 was altered in *pgap3A* whereas GFP-GPI was not, suggesting that trafficking of different GPI-APs may be altered to varying degrees depending on the type of protein and the context.

The N-terminally GFP-tagged PGAP3A and B versions mostly localized at the ER. In contrast, the C-terminally mRFP-tagged version localized mostly in the Golgi. This difference could be explained by the presence of a putative dilysine ER retrieval/retention signal at the C-terminal end of both PGAP3A and B that may be masked by the C-terminal RFP tag. The dilysine signals are known to bind to COPI coat proteins and mediate retrieval of proteins from post-ER compartments to the ER by COPI vesicles (Cosson and Letourneur, 1994; Jackson et al., 2012; Gao et al., 2014). It cannot be ruled out that PGAP3 proteins contain additional sorting signals. In addition, putative sorting signals might be altered by post-translational modifications and/or oligomerization, as it happens with the addition of XFP tags. Therefore, it is difficult to predict the real steady-state localization of PGAP3A and PGAP3B, although the results presented here clearly indicate that they may cycle between the ER and the Golgi apparatus (Supplementary Figure 9). This may explain the delay in transport to the plasma membrane/cell wall of GFP-AGP4 and V-FLA11 in pgap3A mutants. Indeed, PGAP3A and PGAP3B localization correlates with GFP-AGP4 and V-FLA11 ER/Golgi patterns observed in transient expression experiments of *pgap3A* mutants.

*Arabidopsis* contains two PGAP3 isoforms, in contrast to yeast and mammals that contain only one isoform. *pgap3A* did not show any growth alterations under standard growth conditions but it is more sensitive to different stress conditions. In addition, the mutant showed a delay in the trafficking of GPI-APs to the cell surface. On the other hand, *PGAP3B*, but not *PGAP3A*, was able to complement the yeast *per1* mutant. This raises the possibility that PGAP3A and PGAP3B may have different specificities and have not completely redundant functions. In the future, generation of different CRISPR *pgap3B* mutant lines, for example with substitutions/deletion of putative amino acids of the active site, will assist efforts to understand if the roles of these two proteins are distinct or overlapping. It is intriguing that no *PGAP2* gene has been identified yet in *Arabidopsis* (Luschnig and Seifert, 2011). PGAP2, which acts after PGAP3, is involved in GPI anchor reacylation in mammals and there is evidence that PGAP2 and PGAP3 may form a complex. Nevertheless, as two or more PGAP3 isoforms have been identified in most plant species (Supplementary Table 3) (Thomas et al., 2021), it is exciting to think that these isoforms may reflect differences in plant GPI anchors. To address a major gap in knowledge of key importance, it would be essential to solve other plant GPI-AP structures and gain better understanding of plant GPI biology.

## Data Availability Statement

The original contributions presented in the study are included in the article/Supplementary Material, further inquiries can be directed to the corresponding authors.

## Author Contributions

MM, FA, KJ, and AF: conceptualization. CB-S, YM, AF, MM, and FA: investigation. MM and FA: writing – original draft, supervision, project administration, and funding acquisition. MM, FA, KJ, AF, and CB-S: writing – review and editing. All authors contributed to the article and approved the submitted version.

## Conflict of Interest

The authors declare that the research was conducted in the absence of any commercial or financial relationships that could be construed as a potential conflict of interest.

## Publisher’s Note

All claims expressed in this article are solely those of the authors and do not necessarily represent those of their affiliated organizations, or those of the publisher, the editors and the reviewers. Any product that may be evaluated in this article, or claim that may be made by its manufacturer, is not guaranteed or endorsed by the publisher.

## Footnotes

1
https://abrc.osu.edu/

2
http://www.bio-rad.com/

3
www.arabidopsis.org

4
http://signal.salk.edu/cgi-bin/tdnaexpress
