# Supplementary material for: Characterization of Arabidopsis Post-Glycosylphosphatidylinositol Attachment to Proteins Phospholipase 3 Like Genes
Source: Front Plant Sci. 2022 Feb 11;13:817915. doi: 10.3389/fpls.2022.817915 (PMC8874281; doi:10.3389/fpls.2022.817915)
Supplement: Supplementary file 1 [file Data_Sheet_1.PDF]

Characterization of Arabidopsis Post-Glycosylphosphatidylinositol (GPI) Attachment to Proteins phospholipase 3 (PGAP3) like genes

Cesar Bernat-Silvestre[1], Yingxuan Ma[2,3], Kim Johnson[2,3], Alejandro Ferrando[4], Fernando Aniento[1]*†, María Jesús Marcote[1]*†

SUPPLEMENTAL MATERIAL

Supplemental Figure S1. Alignment of the protein sequences of Per1 family members and topology of AtPGAP3A and AtPGAP3B proteins.

Supplemental Figure S2. Developmental expression of Arabidopsis *PGAP3A* and *PGAP3B*.

Supplemental Figure S3. N-terminal and C-terminal PGAP3A and PGAP3B XFP tagged proteins.

Supplemental Figure S4. Phenotypic analysis of *pgap3A* and *pgap3B* mutants

Supplemental Figure S5. Localization of organelle marker proteins in wild-type (Col-0) and *pgap3AB-2*.

Supplemental Figure S6. Localization of GFP-AGP4 and GFP-GPI in wild-type, *pgap3A-1*, *pgap3B-2* and *pgap3AB-2 Arabidopsis* protoplasts.

Supplemental Figure S7. Colocalization of GFP-AGP4 and GFP-GPI in *pgap3AB-2 Arabidopsis* protoplasts.

Supplemental Figure S8. Localization of plasma membrane proteins without a GPI anchor in wild-type, *pgap3A-1*, *pgap3B-2* and *pgap3AB-2* protoplasts.

Supplemental Figure S9. Scheme of GPI anchor lipid remodelling in *Arabidopsis*.

Supplemental Table S1. *pgap3A* and *pgap3B* mutants and PCR primers used for their characterization.

Supplemental Table S2. List of primers used for RT-sqPCR.

Supplamental Table S3. Number of PGAP3 genes found in different animal, plant and fungi species

A

```
Per1p           -MRL--AVVVTLLVHC--FLVTCSPGDNLDEFIDCTYACEYNRRCPNSQINYI-DPETNM  54
HsPGAP3         -MA--GLAARLVLLAGAAALASGSQGDREPVYRDCVLQCEEQNCSGGAL-----------  46
AtPGAP3A        MARNLPWVLLIIVVSCLVSTLEASEGDSDSLYKSCVDQCQKTGCVGDTCFQHCKFSADGK  60
AtPGAP3B        -MAVHYWTALFLLLPCLFCISNASAGDADPDYRTCVSECEISGCVGQLCFPQCNSSSDG-  58
                       .   :::         * **    :  *.  *:

Per1p           FHDIEFFDTPPLYSKLLFWDCISDCDYQCQHIITRWRIDEEEEIYQFHGKWPFLRVLGTQ  114
HsPGAP3         ---NHFRSRQPIYMSLAGWTCRDDCKYECMWVTVGLYLQEGHKVPQFHGKWPFSRFLFFQ  103
AtPGAP3A        AIDGPWYMQEPLYLRWKQWDCQSDCQYECMMTREEERKRNGERPTKYFGKWPLKHVYGIQ  120
AtPGAP3B        ---GPWYIQEPLYLQWKKWGCQGDCRYQCMVNRETERETLGQAPVKYHGKWPFKRVLGIQ  115
                     :    *:*     * * .** *:*            .   ::.****: :.   *

Per1p           EFFSTIFSIGNFIPHYKGFVKFSRIIREEGDRRRKNSRSILIWN--YLYVTVAGMLAWTA  172
HsPGAP3         EPASAVASFLN------GLASLVMLCRYRTFVPASS-PMYH----TCVAFAWVSLNAWFW  152
AtPGAP3A        EPVSVAFSALDLAMQFQGWVSYFILVYYKLPLQPNR-KTYYEYNGIVHIYAIIVMNSLFW  179
AtPGAP3B        EPASVAFSVLNLAMHFHGWLSFFIMIYYKLPLKQDR-TAYYEYVGLWHIYGLLSMNSWFW  174
                *  *.  *  :      *  .   :   .     .                   : :

Per1p           SSVFHCRDLIITEKLDYFFAGLTVLTGFHAIFARMTSMFL-YPKIAQAFTASVAAIFALH  231
HsPGAP3         STVFHTRDTDLTEKMDYFCAST--VILHSIYLCCVRTVGLQHPAVVSAFRALLLLMLTVH  210
AtPGAP3A        SSICHSRDVELTERLDYSSATV--LAGFSLILAILRSFSIQDQSVKIMVTAPILAVVATH  237
AtPGAP3B        SAVFHSRDVDLTERLDYSSAVA--ILGFSLILAILRTFDIRVEAARVMVSAPILAFVTTH  232
                *:: * **  :**::**  *    :  .   :. : :. :        . * :  ..: *

Per1p           ILRL-YVDWSYTYNMRFNIFFGVLQYILLIMLSCQNYHALQKQKLMGEFKKTAYSSFKRQ  290
HsPGAP3         VSYLSLIRFDYGYNLVANVAIGLVNVVWWLAWCLWNQRRLPH------------------  252
AtPGAP3A        ILYLNFYNLDEGLHWKVIFGIGGIELVVWGLWAALTSHP--S------------------  277
AtPGAP3B        ILYINFYKLDYGWNMIVCVAMGVSQLFLWARWAAVSSHP--S------------------  272
                :  :     .   :    . :*  : .     .  . :

Per1p           IFKLCVIPILLVIVTTMAMSLELFDFFSYEWQIDAHALWHLCTIWPSWVLYDFFLEDYAY  350
HsPGAP3         VRKC----VVVVLLLQGLSLLELLDFPPLFWVLDAHAIWHISTIPVHVLFFSFLEDDSLY  308
AtPGAP3A        KWKL----RAFLISSILTLCLRMFDFPPYKGYIDAHALWRGAGIPLSYLWWSFVCDDAVF  333
AtPGAP3B        NWKL----WVVVIAGGLAMLLEIYDFPPYEGYFDAHSIWHAATIPLTILWWSFIRDDAEF  328
                  *       .::       *.: **      :***::*: . *    : :.*. :*  :

Per1p           WGNRQLY-------      357
HsPGAP3         LLKESEDKFKLD--      320
AtPGAP3A        RTTVNLKKSK----      343
AtPGAP3B        RTSSLLKKTKTKAK      342
                  .
```

B

AtPGAP3A

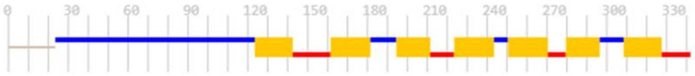

AtPGAP3B

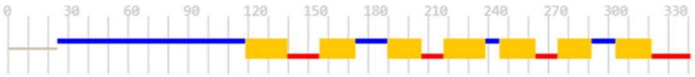

Supplemental Figure S1. A. Alignment of the protein sequences of Per1 family members: Per1p (*S. cerevisiae*), HsPGAP3 (Human), AtPGAP3A (At5g62130.1) (*A. thaliana*) and AtPGAP3B (At1g16560.1) (*A. thaliana*). ClustalW software was used to generate the alignment. Conserved putative active site residues and putative dilysine ER retrieval/retention signals present at the C-terminal end are shown in grey and yellow, respectively. PGAP3A and PGAP3B contain a KKxx and a KxKxx dilysine motif, respectively. A KxKxx motif is also present in HsPGAP3. **B.** Topology of AtPGAP3A and AtPGAP3B proteins as predicted by TMHMM method. Results obtained by the CCTOP web server (Dobson et al., 2015). Colors are based on the putative localization: blue, red and yellow for luminal, cytoplasmic and membrane regions, respectively. Seven transmembrane domains are predicted, as occurs in other members of the Per1 family.

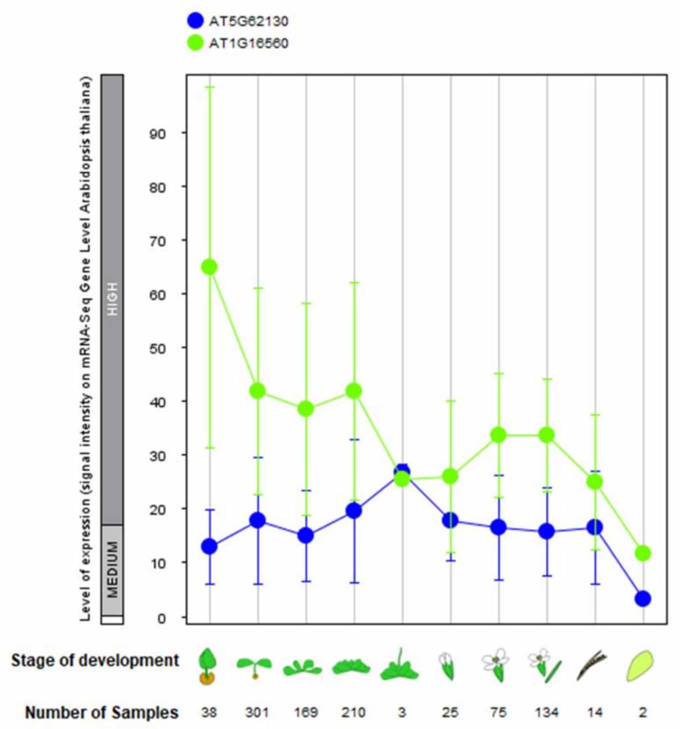

Supplemental Figure S2. Developmental expression of Arabidopsis *PGAP3A* and *PGAP3B*. The level of expression of *PGAP3A* (At5g62130, blue) and *PGAP3B* (At1g16560, green) at 10 stages of development including seedlings, rosette leaves, floral organs and siliques. “HIGH”, “MEDIUM”, and “LOW” expression were calculated by RNA-seq assay and showed *PGAP3B* is highly expressed at early stages of development and overall has higher levels of expression than P*GAP3A*. The number of samples indicates RNA-seq gene expression data collected by GENEVESTIGATOR (www.genevestigator.com). Error bars represent standard deviation.

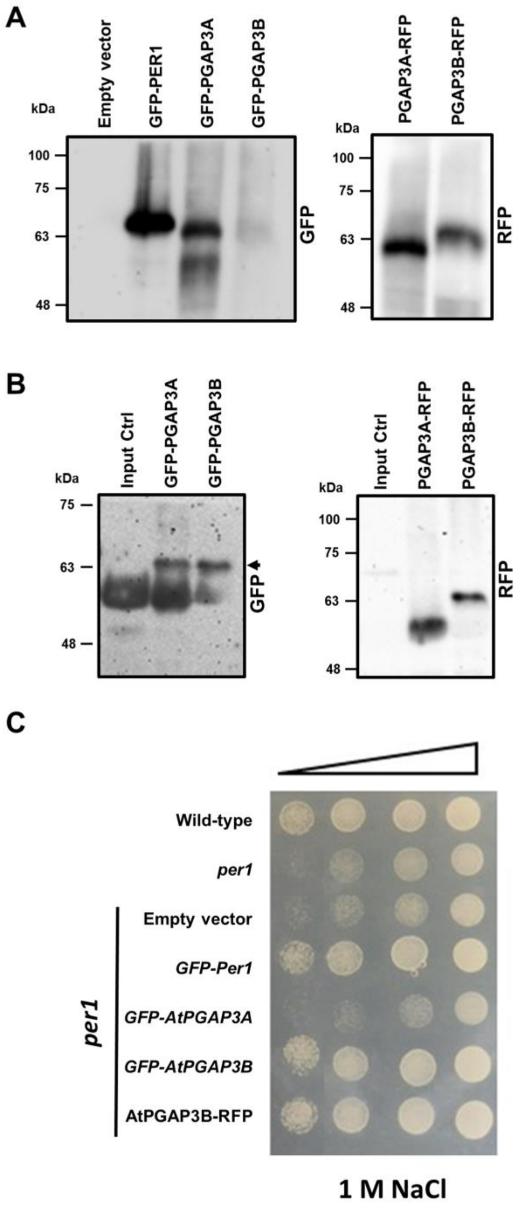

Supplemental Figure S3. N-terminal and C-terminal PGAP3A and PGAP3B XFP tagged proteins. A. Western blot showing detection of PGAP3A, PGAP3B and Per1 proteins tagged with XFP expressed in yeast. 20 ml of yeast culture were loaded in each lane. αGFP (left panel) and αRFP (right panel) antibodies were used **B.** Western blot showing detection of PGAP3A and PGAP3B proteins tagged with XFP and expressed in *N. benthamiana* leaves. 12 mg of total proteins were loaded in each lane. αGFP (left panel) and αRFP (right panel) antibodies were used. A difference in molecular weight between PGAP3A-RFP and PGAP3B-RFP was observed in both yeast and *N. benthamiana* and may indicate post-translational modification of PGAP3B. **C.** Similar to GFP-Per1, N- and C-terminal XFP-tagged AtPGAP3B complemented the sensitivity of *per1* yeast mutant (Y15769) to 1M NaCl whereas GFP-AtPGAP3A did not. Wild-type and mutant Y15768 strains (*per1*) were grown for 2 days in synthetic medium supplemented with the required amino acids and spotted in YPD medium supplemented with 1 M NaCl, as indicated. Growth was scored after 5 days. The triangle represents an increase in yeast concentration from left to right.

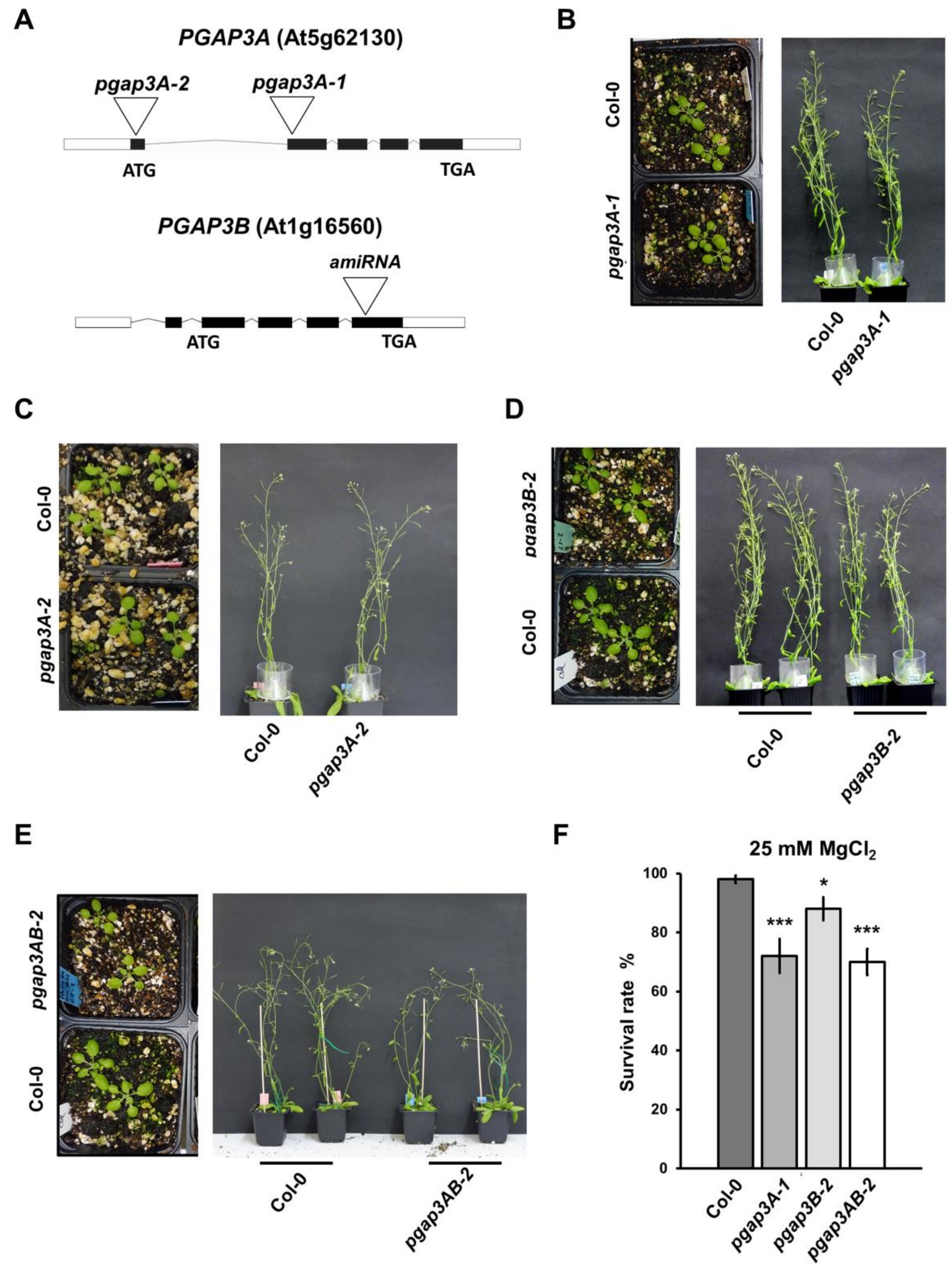

Supplemental Figure S4. Phenotypic analysis of *pgap3A* and *pgap3B* mutants. A. Diagram of the *PGAP3A* (At5g62130.1) and *PGAP3B* (At1g16560) genes and localization of the T-DNA insertion in *pgap3A-1 and pgap3A-2* mutants and *amiRNA-PGAP3B* target in *PGAP3B* (triangles). Black boxes represent coding regions and white boxes represent 5′ UTR and 3′ UTR regions. **B-C.** Left panel, 15 day-old plants and right panel, 35 day-old plants of wild-type (Col-0) and the *pgap3A-1*(B) and *pgap3A-2* (C) mutants. **D-E**. Left panel, 15 day-old plants and right panel, 30 day-old plants of wild-type (Col-0), *pgap3B-2* (D) and *pgap3AB-2*(E). *pgap3B* and *pgap3AB* plants smaller than wild-type plants are occasionally observed but they have the same *PGAP3A/B* mRNA levels as *pgap3B* and *pgap3AB* plants that have the same height than wild-type plants. **F.** Wild-type (Col-0), *pgap3A-1*, *pgap3B-2* and *pgap3AB-2* were grown on 0.5 × MS as a control and 0.5 × MS supplemented with 25 mM $MgCl_2$. The percentage of seedling survival was calculated after 18 days and data are mean ± s.e.m. (n =20) of five independent experiments. Statistical significance: *$p < 0.05$; **$p < 0.01$; ***$p < 0.001$.

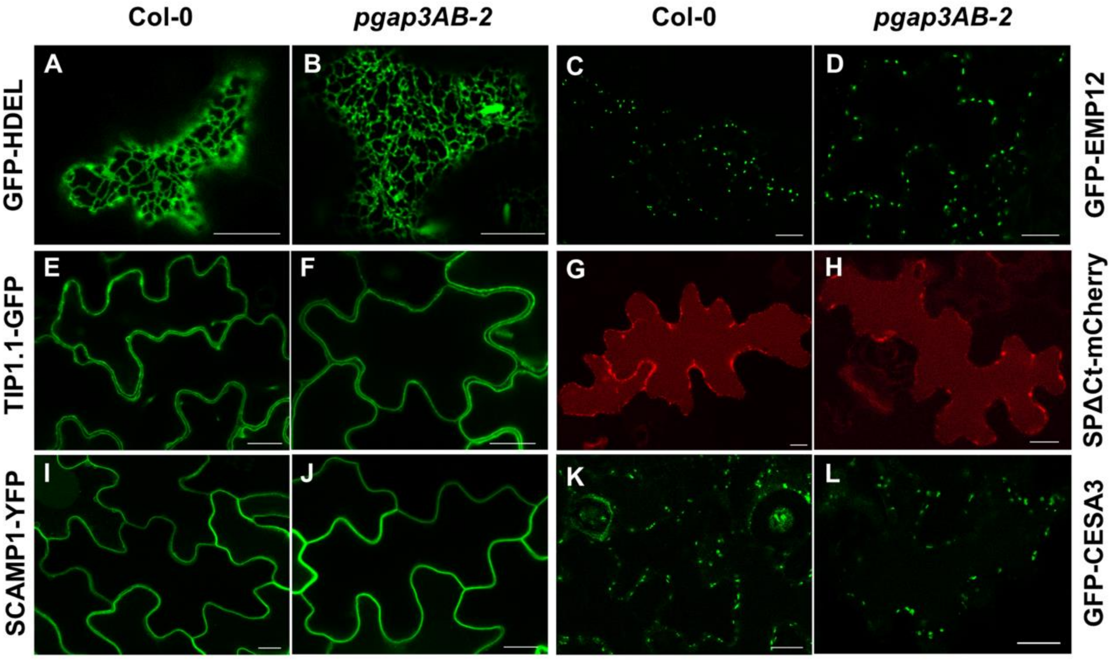

Supplemental Figure S5. Localization of organelle marker proteins in wild-type (Col-0) and *pgap3AB-2*. Transient expression in *Arabidopsis* seedlings of different organelle marker proteins, including GFP-HDEL (endoplasmic reticulum) (A-B), GFP-EMP12 (Golgi apparatus) (C-D), TIP1.1-GFP (tonoplast) (E-F), SPΔCt-mCherry (vacuole lumen) (G-H), SCAMP1-YFP (plasma membrane) (I-J) or GFP-CESA3 (*trans*-Golgi network (TGN)/plasma membrane) (K-L) showed no difference between wild-type (Col-0) and *pgap3AB-2* mutants. Scale bars = 10 µm.

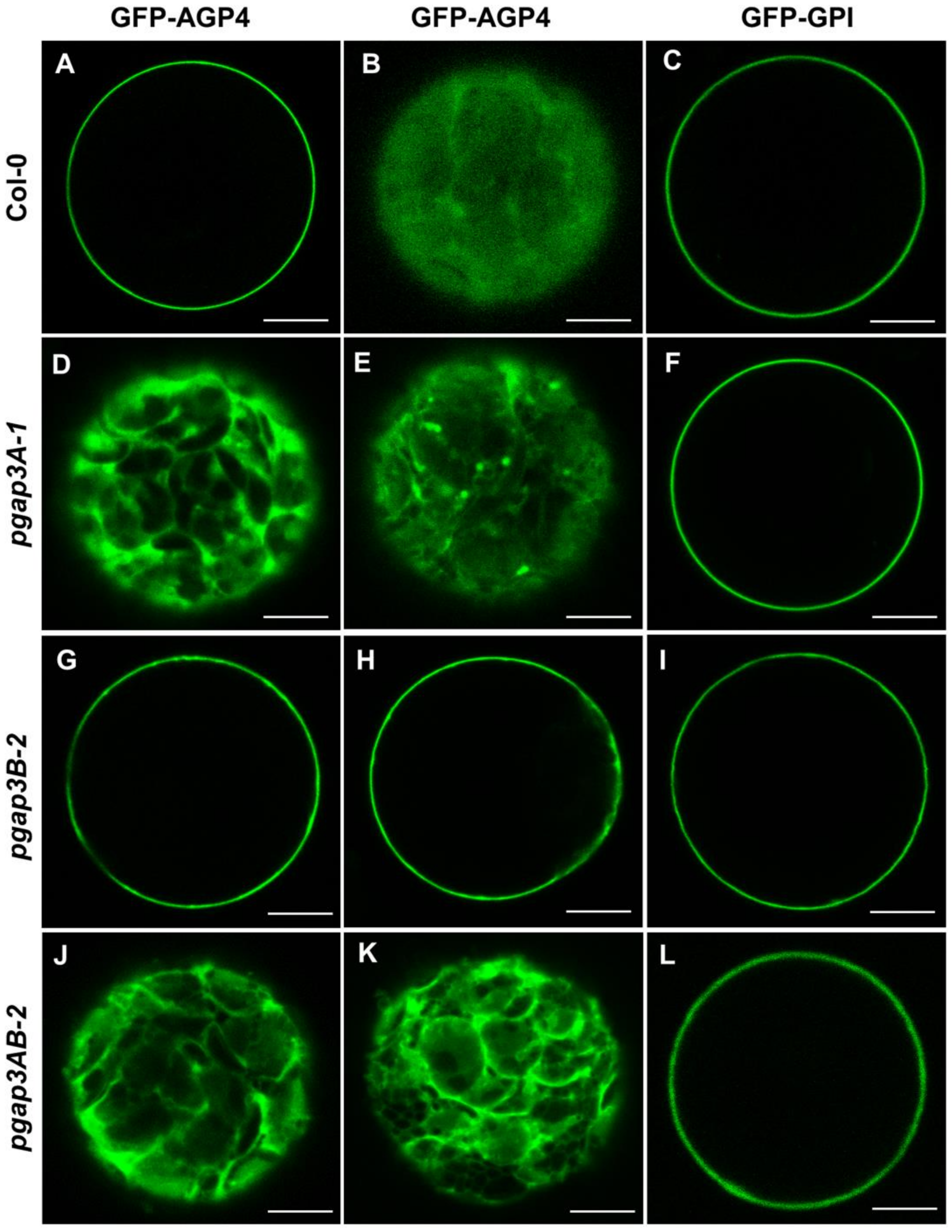

Supplemental Figure S6. Localization of GFP-AGP4 and GFP-GPI in wild-type, *pgap3A-1*, *pgap3B-2* and *pgap3AB-2 Arabidopsis* protoplasts. Transient expression in wild-type (A-C) and *pgap3A-1* (D-F), *pgap3B-2* (G-I) and *pgap3AB-2* (J-L) *Arabidopsis* protoplasts. GFP-AGP4 (A, B) and GFP-GPI (C) localized to the plasma membrane in wild-type protoplasts. In contrast, GFP-AGP4 showed an ER-like localization and a Golgi-like localization pattern in *pgap3A-1* (D-E) and *pgap3AB-2* (J-K), while GFP-GPI localized to the plasma membrane (F and L). GFP-AGP4 and GFP-GPI showed a typical plasma membrane localization in *pgap3B-2* mutant (G-I). Scale bars = 10 µm.

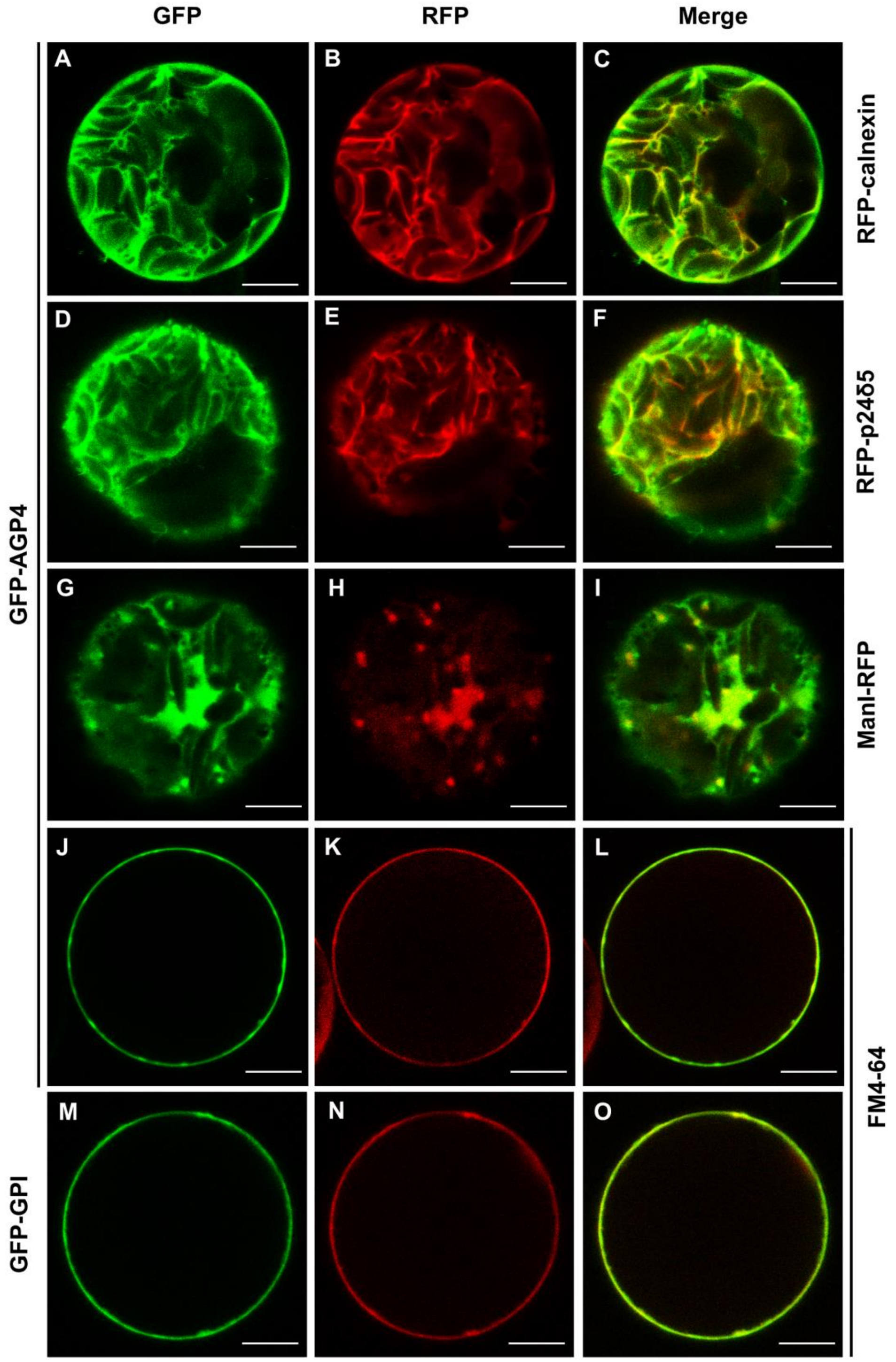

Supplemental Figure S7. Colocalization of GFP-AGP4 and GFP-GPI in *pgap3AB-2 Arabidopsis* protoplasts. A-I. Coexpression of GFP-AGP4 (A, D, G) with the ER markers RFP-calnexin (B) and RFP-p24δ5 (E) or with the Golgi marker ManI-RFP (H) (see merged images in C, F and I). **J-L**. Colocalization of GFP-AGP4 (J) and GFP-GPI (M) with the FM dye FMA-64 (K, N) (see merged images in L and O). Scale bars = 10 µm.

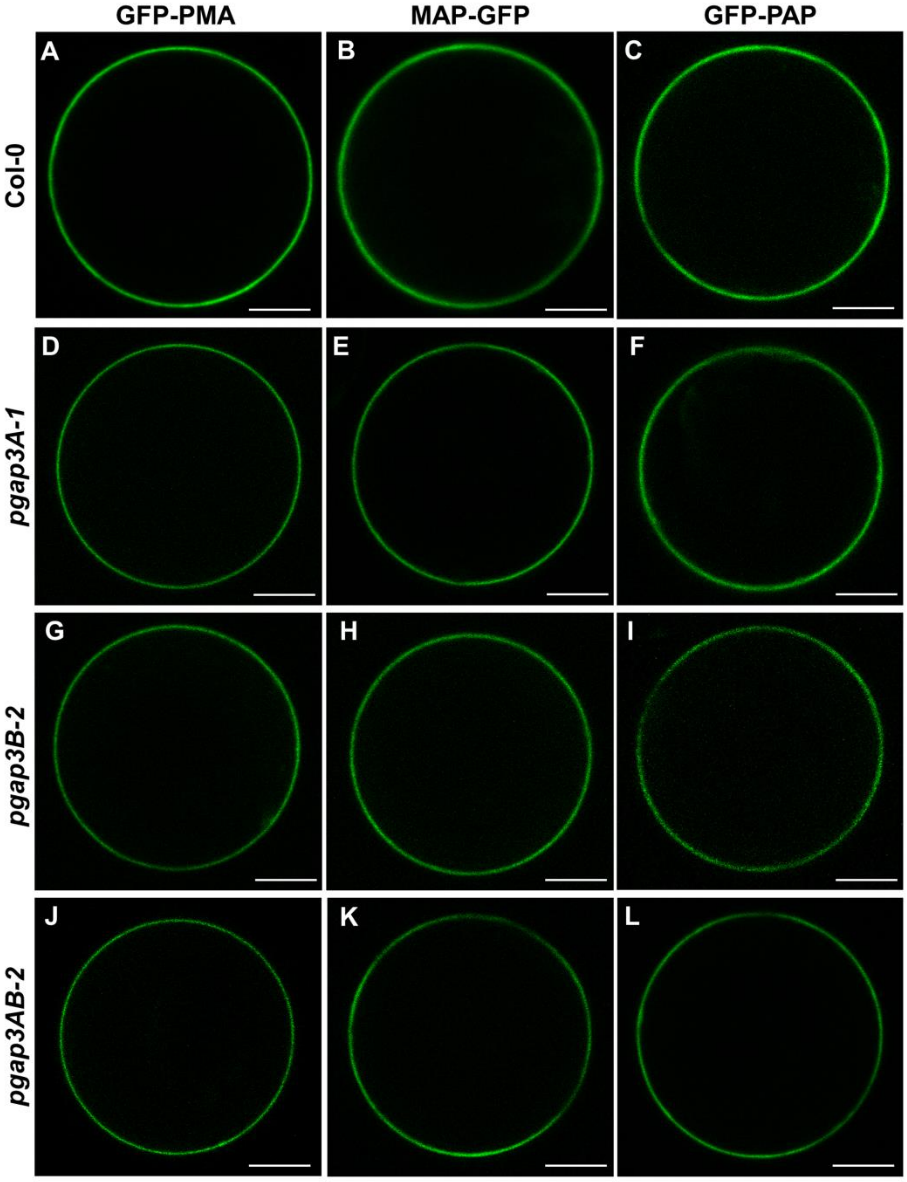

Supplemental Figure S8. Localization of plasma membrane proteins without a GPI anchor in wild-type, *pgap3A-1*, *pgap3B-2* and *pgap3AB-2* protoplasts. GFP-PMA (A, D, G, J), MAP-GFP (B, E, H, K) and GFP-PAP (C, F, I, L) localized mainly to the plasma membrane in protoplasts from wild type (A to C), *pgap3A-1* (D to F), *pgap3B-2* (G to I) and *pgap3AB-2* (J-L). Scale bars = 10 µm.

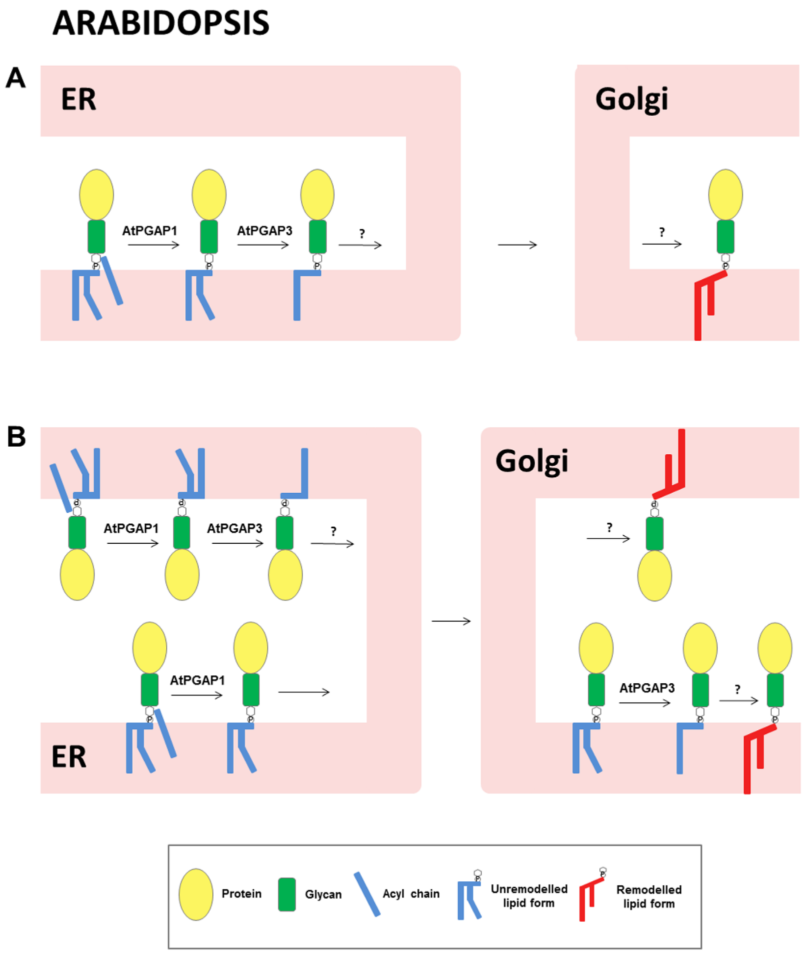

Supplemental Figure S9. Scheme of GPI anchor lipid remodelling in *Arabidopsis*. Lipid remodelling is initiated at the ER by AtPGAP1, an inositol deacylase that removes the acyl chain at the 2-position of the inositol ring (Bernat-Silvestre et al., 2021). Next, AtPGAP3(A/B) may be involved in lipid remodeling of phosphatidylinositol, like in mammals and yeast. As AtPGAP3(A/B) contain a putative dilysine ER retrieval signal at the C-terminus, they may cycle between ER and Golgi and thus two scenarios can be contemplated. A. AtPGAP3(A/B) localise to the ER and lipid remodelling occurs at the ER. B. AtPGAP3(A/B) cycle between ER and Golgi. In this case, AtPGAP3 could act either at the ER and/or Golgi.

Gene	Mutant	Genotyping primers		RT-sqPCR primers
		**T-DNA insertion**	**Wild-type allele**	
AT5G62130 (*pgap3A-1*)	SALK_039375	LBb1/RPPGAP3A	RPPGAP3A/LPPGAP3A	RPPGAP3A/LPPGAP3A
AT5G62130 (pgap3*A-2*)	SALK_069053	LBb1/NRPPGAP3A	NRPPGAP3A/NLPPGAP3A	RPPGAP3A/LPPGAP3A
AT1G16560	*amiR-pgap3B*	ami3/ami5	PGAP3BR/ PGAP3BF	PGAP3BR/ PGAP3BF

Supplemental Table S1. *pgap3A* and *pgap3B* mutants and PCR primers used for their characterization. Genotyping primers used for the identification of the mutants and RT-sqPCR primers used to verify the absence of mRNA in the mutants.

Primer	Gene	Sequence (5’ → 3’)	Tm (ºC)
RPPGAP3A	AT5G62130	AATGTCAGAAAACTGGATGCG	60
LPPGAP3A	AT5G62130	TGCTCGAGATCAAGAAAGCTC	62
NRPGAP3A	AT5G62130	CATCCACCTGTCTCCGTAATTTGA	57
NLPGAP3A	AT5G62130	TGCTCGAGATCAAGAAAGCTCTTAG	57
PGAP3BR	AT1G16560	GGATCCAAATCCTTGTAAAACTTAGC	63
PGAP3BF	AT1G16560	GTCAAGGTGATTGCCGTTAT	56
Housekeeping genes			
A5	*ACT-7*	GGATCCAAATGGCCGATGGTGAGG	69
A3	*ACT-7*	GGAAAACTCACCACCACGAACCAG	67
Insertion			
LBb1	T-DNA	GGATCCGCGTGGACCGCTTGCTGCAACT	76
ami3	*amiRNA*	GGATCCGCAATTAACCCTCACTA	63
ami5	*amiRNA*	ATATAAGGAAGTTCATTTCATTTGGAG	61

Supplemental Table S2. List of primers used for RT-sqPCR.

**Number of PGAP3 genes**	**Animal (51 species)**	**Plant (38 species)**	**Fungi (13 species)**
1	50	2 (*Zostera marina* and *Amborella trichopoda*)	13
2	1 (*Strongylocentrotus purpuratus*)	12	
3		11	
4		4	
>4		9	

Supplemental Table S3. Number of PGAP3 genes found in different animal, plant and fungi species (http://www.pantherdb.org/panther/family.do?clsAccession=PTHR13148:SF0) (Thomas et al., 2021).

Thomas PD, Ebert D, Muruganujan A, Mushayahama T, Albou LP, Mi H. (2021) PANTHER: making genome-scale phylogenetics accessible to all. Protein Sci. 2021 Oct 30. doi: 10.1002/pro.4218.
